# Emotional intelligence and clinical competence among nursing interns: a structural equation modeling study

**DOI:** 10.3389/fmed.2026.1816502

**Published:** 2026-05-22

**Authors:** Sitah S. Alshutwi, Nouf Afit Aldhafeeri, Latifah Alenezi

**Affiliations:** 1 College of Nursing, King Saud Bin Abdulaziz University for Health Sciences, Riyadh, Saudi Arabia; 2 King Abdullah International Medical Research Center, Riyadh, Saudi Arabia; 3 Ministry of the National Guard-Health Affairs, Riyadh, Saudi Arabia

**Keywords:** clinical practice, emotional intelligence, nursing interns, quality nursing care, workforce

## Abstract

**Background:**

Emotional intelligence (EI) has been recognized as a key psychological resource for managing emotions and coping with highly stressful healthcare environments, which may influence clinical competence. Nursing internship is a critical period for developing clinical skills amid increasing expectations for interns. Despite growing evidence, there remains a notable lack of empirical evidence examining whether emotional intelligence influences the clinical competence of nursing interns in Saudi Arabia. This study aimed to examine the association of emotional intelligence with the clinical competence among nursing interns.

**Method:**

A descriptive cross-sectional, correlational design using structural equation modelling was used to test a hypothesized model. A convenience sample of 181 nursing interns was recruited from a governmental hospital in Riyadh, Saudi Arabia. Eligible participants were interns currently enrolled in the internship program; those on extended leave, unable to complete the assessment, or with conditions affecting clinical performance were excluded. Data were collected using an online survey. Emotional intelligence was measured using the Wong and Law Emotional Intelligence Scale (WLEIS), and clinical competence was assessed using the Holistic Clinical Assessment Tool (HCAT). Data was analyzed using descriptive statistics and structural equation modeling (SEM) to examine direct and indirect relationships between variables.

**Results:**

The mean score for EI was 5.0 ± 1 and for clinical competence was 3.1 ± 0.9. In the structural model, EI (WLEIS) had a significant positive effect on the clinical competence (HCAT) with a standardized coefficient of 0.62. Age was positively associated with all subdomains of clinical competence and with overall clinical competence (HCAT) (standardized coefficient = 0.25), while GPA showed a significant association with EI (WLEIS) (standardized coefficient = 0.50).

**Conclusion:**

EI plays a crucial role in enhancing the clinical competence of nursing interns. Hence, integrating emotional intelligence into the nursing curriculum and providing emotional intelligence training for nursing interns are recommended to enhance their emotional management and clinical competence.

## Introduction

The stressful nature of healthcare environments can adversely affect the mental and emotional well-being of nurses. In recent years, EI has emerged as a crucial psychological resource, demonstrating a positive influence on mental health. Within the dynamic and emotionally charged healthcare environment, EI has gained increasing attention. It is now considered a critical component of effective nursing practice. Nurses are routinely exposed to a wide spectrum of emotions, including human pain and suffering. The development and application of EI skills serve as an effective strategy for preserving their emotional and mental well-being (1).

EI was originally defined as a form of social intelligence that involves the ability to monitor one’s own and others’ emotions, to discriminate among them, and to use this information to guide one’s thinking and behavior (2). EI can be conceptualized and measured across four key dimensions. The first is Self-Emotion Appraisal (SEA), which refers to an individual’s ability to recognize, understand, and naturally express their own emotions. The second is Others’ Emotion Appraisal (OEA), involving the capacity to accurately perceive and interpret the emotional states of others. The third dimension, Regulation of Emotion (ROE), refers to the ability to manage and control emotional responses. This facilitates faster recovery from psychological stress. Finally, Use of Emotion (UOE) pertains to the ability to harness and channel emotions toward constructive outcomes, such as enhancing motivation, guiding problem-solving, and improving overall performance (3).

EI has been shown to influence various aspects of nursing practice, including ethical decision-making and critical thinking. Emotional intelligence may facilitate the integration of evidence and knowledge into clinical practice, thereby enhancing care quality and improving patient outcomes (4). Further, EI has been shown to be significantly and positively correlated with overall clinical competence. This suggests that individuals with higher levels of EI tend to perform better in clinical settings (5).

clinical competence is an essential competency for every nurse. Clinical competence is a multidimensional concept that integrates knowledge, skills, judgment, behaviors, and professional attitudes in nursing practice (6). The ability of a nurse to practice is defined, following Wu et al. (7), as clinical competence. This is a multidimensional construct encompassing the knowledge, skills, and professional attributes required to deliver safe, effective, and high-quality patient care. Existing literature demonstrates that EI plays a pivotal role in the nursing profession. It contributes to the development of therapeutic nurse–patient relationships, the delivery of high-quality care, and the effective use of the nurse’s consultative role within the healthcare team (8).

A study conducted in mainland China found that the EI of nursing students is a significant predictor of their clinical competence. The findings suggest that higher levels of EI are associated with improved abilities in providing high quality nursing care; the scores were positively correlated with clinical competence (R = 0.534, *p* < 0.05). This indicates that individuals with higher EI tend to demonstrate stronger clinical competence (5). Another study conducted in Saudi Arabia found a significant positive relationship between emotional intelligence and nurses’ work performance (R^2^ = 0.657, *p* < 0.001) (9).

Unlike experienced nurses who have established relatively stable careers, nursing interns go through a difficult transition from nursing student to novice nurse (10). Nurse interns often face significant challenges, including unfamiliarity with clinical environments, limited practical skills, and insufficient theoretical knowledge and communication abilities (11). These gaps may compel nurse interns to rely heavily on emotion regulation strategies to effectively navigate the daily demands of clinical practice. Consequently, understanding the role of emotional intelligence in influencing the clinical competence of nurse interns has become an important focus of research (5).

In Saudi Arabia, the nursing internship program represents a pivotal component of nursing education, designed to bridge the gap between theoretical learning and clinical practice. The internship is mandated by the Saudi Commission for Health Specialties (SCFHS), which conducts the licensure process for graduates. Completion of the internship and the certificate is required for eligibility for professional registration and licensure. These are necessary for employment in public and private healthcare institutions across the country (12). This regulatory framework ensures that nurse interns not only meet academic standards but also demonstrate competence in real-world clinical environments.

During the internship program, nursing interns often experience high levels of stress and emotional challenges (11). Developing critical emotional skills is essential for nurse interns as they transition into the workforce. This need is underscored by increasing demands from health services for nursing graduates to be work-ready at the onset of their careers (13). The nursing internship represents a critical period for nursing graduates to acquire clinical skills and develop their clinical competence. EI plays a central role in clinical practice. Nurses must manage their emotions while providing psychological support to patients and their families (14). In previous studies, it has been indicated that there is a positive significant relationship between EI and problem-solving skills (15), critical thinking (16), and empathy (17). These are essential skills that nurse interns need. Furthermore, EI has been shown to predict nursing students’ success in clinical settings (18). Therefore, assessing the EI of nursing interns and investigating its relationship with clinical competence is significant.

Although some studies have explored the relationship between EI and clinical competence among nursing interns, the specific pathways through which EI affects clinical competence remain unclear. This includes the relative influence of its four branches (Self-Emotion Appraisal, Others’ Emotion Appraisal, Use of Emotion, and Regulation of Emotion). Furthermore, the role of demographic factors in this process requires further investigation.

Understanding the influence of EI on nursing interns’ clinical competence is crucial within the evolving Saudi healthcare system. This system is undergoing fundamental reforms aligned with Saudi Arabia Vision 2030. As the system moves toward care models that emphasize interdisciplinary collaboration, nurses’ ability to manage emotions, communicate, and adapt becomes increasingly critical. This is essential for ensuring quality care and successful implementation of improvement strategies (19).

Despite growing evidence on the positive role of emotional intelligence in nursing practice, there remains a notable lack of empirical research examining its relationship with the clinical competence of nursing interns within the Saudi Arabian context. Additionally, the specific mechanisms through which EI influences clinical competence particularly the relative contribution of its four dimensions and the role of demographic factors are not well understood. Most existing studies rely on simple correlational analyses, with limited use of advanced modelling approaches to explore direct and indirect relationships. Therefore, further investigation is needed to provide a comprehensive understanding of how EI shapes clinical practice during the internship period.

Accordingly, this study was designed to address these gaps by clearly defining its objectives and examining the proposed relationships. The present study aimed to examine the association between EI and clinical competence of nursing interns in Saudi Arabia. Specifically, this study sought to assess the level of EI and clinical competence among nursing interns and to explore the relationship between these variables. In addition, this study aimed to determine the extent to which EI predicts clinical competence, while also examining the influence of selected demographic factors, such as age and academic performance, on both EI and clinical competence. Furthermore, this study investigated both the direct and indirect relationships between EI and clinical competence using structural equation modelling.

## Materials and methods

### Study design

A descriptive cross-sectional, correlational design using structural equation modelling was used to examine the path relationship of EI with the clinical competence of nursing interns.

### Study subjects

The study was conducted in a governmental hospital located in Riyadh city including nursing interns from the College of Nursing. Inclusion criteria include nursing interns who have started internship program and are willing to participate in the study. Exclusion criteria nursing interns on long leaves such as maternity leave extended sick leave or have suspended their internship. Interns presenting with severe or unstable psychiatric conditions (e.g., severe anxiety or major depressive disorder), as well as those with neurological or medical conditions that could compromise clinical performance, were excluded from the study. In addition, individuals who have recently undergone intensive EI training were not be eligible for participation.

### Sample size

The Sample size was calculated to estimate the mean scores of EI (WLEIS) and clinical competence (HCAT) with adequate precision. Using Mthimunye and Daniel’s (20) approach for estimating a population mean, assuming a standard deviation of 1.0, a 95% confidence level, and a margin of error of 0.15, the minimum required sample size was 171. The sample achieved was also adequate for the planned correlational analyses and structural equation modeling, where WLEIS and HCAT were modeled as latent variables indicated by their respective domain scores. To determine an appropriate sample size for the structural equation modeling (SEM) analysis, we performed a series of simulations using the lavaan package in R. Data were simulated based on a predefined population model across a range of sample sizes, from 100 to 300, increasing by increments of 50. For each sample size, 100 simulations were executed, and model fit was evaluated using the Comparative Fit Index (CFI), Tucker-Lewis Index (TLI), Root Mean Square Error of Approximation (RMSEA), and Standardized Root Mean Square Residual (SRMR). The simulation results indicated that a sample size of 150 provided satisfactory fit indices, with CFI and TLI values above 0.95, RMSEA well below 0.05, and an SRMR of 0.07, all suggesting a good fit to the data. To enhance representativeness, a larger sample was targeted, and 181 participants were ultimately included in the analysis.

### Sampling technique

A non-random convenience sampling technique was used in this study. Nursing interns were selected using a convenience sampling method as it provides an efficient and cost-effective approach to recruiting readily available participants (21).

### Data collection methods, instruments used, measurements

After obtaining ethical approval, data were collected online using Microsoft Forms platform. The online link included general information about the study, such as the aim and inclusion and exclusion criteria, informed consent, and study instruments. The survey consisted of three parts:

Part I: Social-demographic data including GPA, English score, age, marital status, internship duration, and previous knowledge about emotional intelligence.
Part II: clinical competence was measured with the Holistic Clinical Assessment Tool (HCAT) developed by Singaporean scholars (7). The HCAT includes four dimensions: specialty, legal and ethical practice, clinical care, leadership and nursing management, and professional development. Cronbach *α* coefficient, test–retest reliability and average content validity of the scale reported as 0.965, 0.928 and 0.972, respectively. The scale included 36 items, with a 4-point Likert scoring method (1, 2, 3, and 4 points = unqualified, qualified, skilled and excellent, respectively). The scores ranged from 36 to 144; where higher scores indicate higher levels of clinical competence. The total score ranged between 36 and 144 points, and the higher the direct total score of all items, the higher the holistic clinical competence. The Holistic Clinical Assessment Tool (HCAT) has been internationally validated but has limited application in the Gulf region. However, its use in this study is appropriate, as it is administered in English, which is the official language of nursing education and clinical practice in Saudi Arabia In this study, internal consistency for HCAT was high, with Cronbach’s alpha values ranging from 0.96 to 0.99, reflecting strong reliability across the scale.
Part III: The 16-item Wong and Law Emotional Intelligence Scale (WLEIS) developed by Wong and Law (3) was used. This scale includes four dimensions: Self-Emotion Appraisal (SEA), Others’ Emotion Appraisal (OEA), Use of Emotion (UOE), Regulation of Emotion (ROE). Responses to the items were coded as follows: 1 = totally disagree, 2 = disagree, 3 = somewhat disagree, 4 = neutral, 5 = somewhat agree, 6 = agree, and 7 = totally agree. Higher scores were indicative of better EI Reliability, the alpha coefficients of the four dimensions were 0.89, 0.88, 0.76, and 0.85 (3). The WLEIS has been used in previous research in Saudi Arabia to assess emotional intelligence among nursing students (22). Its administration in English aligns with the official language of nursing education and clinical practice in the country, supporting its applicability in this study and showed a Cronbach’s alpha ranging from 0.96 to 0.98, confirming strong reliability.

#### Ethical considerations

Approval was obtained from King Abdullah International Medical Research Center (KAIMRC), Riyadh, Saudi Arabia (IRB/307/24, NRC24R/070/02). The researchers followed all ethical considerations during the conduct of the study. Informed consent was obtained online from participants before collecting the data. The participants were assured that their participation was kept confidential. No identifiers were collected, and all data were kept in a secure place.

### Statistical analysis

All analyses were performed using R software version 4.2.2. Continuous variables, such as age, GPA, and average scores for WLEIS and HCAT (with their domains), were summarized with means and standard deviations. Categorical variables, including marital status, internship duration (greater than 8 months or not), and prior knowledge of EI, were summarized using frequencies and percentages. Univariate analyses were conducted to examine associations between participants’ characteristics and both the average domain scores and overall scores of WLEIS and HCAT. Associations between categorical variables and scores were assessed using t-tests, while relationships between numerical variables (age and GPA) and scores were evaluated using Pearson correlation tests.

A structural equation model was implemented using the “lavaan” package. The average subscale scores for WLEIS and HCAT acted as indicators for their respective latent variables in the measurement models. The structural model assessed the association of the latent WLEIS variable and the latent HCAT variable, along with the effects of participant characteristics that showed significant associations with either HCAT or WLEIS in the exploratory univariate analyses. A significance level of 0.05 was applied in all analyses.

## Results

### Participants’ characteristics

Among the study sample (*N* = 181), the mean age of participants was 22.6 (*SD* = ±1.3) years, and the average GPA was 3.8 (*SD* = ± 0.6). Most participants were single (89.0%), had completed an internship duration of more than 8 months (81.2%, *n* = 147), and lacked previous knowledge on EI (72.9%, *n* = 132) (Table 1).

**Table 1:** Characteristics of the study sample (*N* = 181).

Characteristics	M ± SD/*n* (%)
Age	22.6 ± 1.3
GPA	3.8 ± 0.6
Marital status	
Married	20 (11.0)
Single	161 (89.0)
Internship duration	
8 months	34 (18.8)
More than 8 months	147 (81.2)
Previous knowledge	
No	132 (72.9)
Yes	49 (27.1)

Cell values represent frequency and percentage *n*(%) or mean and standard deviation (M ± SD).

### Wong and law emotional intelligence scale (WLEIS) and holistic clinical assessment tool (HCAT)

The average total score of EI among intern students was 5.0 ± 1.6, indicating a moderate to high level of perceived EI across the sample, while the average total score of clinical competence of intern students was 3.1 ± 0.9, indicating a skilled level of competency across domains (Table 2).

**Table 2:** The EI and clinical competence scales.

EI subscales	M ± SD
Self-emotion appraisal (SEA)	5.1 ± 1.7
Others’ emotion appraisal (OEA)	5.1 ± 1.6
Use of emotion (UOE)	5.0 ± 1.7
Regulation of emotion (ROE)	4.8 ± 1.7
Total EI	5.0 ± 1.6
Clinical competence subscales	
Professional, legal and ethical nursing practice (PLENP)	3.1 ± 0.9
Management of care (MC)	3.1 ± 0.9
Leadership & nursing management (LNM)	3.1 ± 0.9
Professional development (PD)	3.1 ± 0.9
Total clinical competence	3.1 ± 0.9

Cell values represent mean ± standard deviation (M ± SD).

### Univariate analyses for the associations between participant characteristics and both the average domain scores and overall scores of EI and clinical competence

Significant associations were found between GPA and all domains of EI, with higher GPA being positively correlated. Age also showed a significant positive correlation with the clinical competence domains and the total score. Additionally, internship duration of more than 8 months was significantly associated with higher scores in the professional, legal and ethical nursing practice (PLENP) and leadership and nursing management (LNM) domains of clinical competence, and previous knowledge was positively associated with the professional, legal and ethical nursing practice (PLENP) domain of clinical competence (Table 3).

**Table 3:** Univariate analyses for the association between participants’ characteristics and the average scores of the WLEIS, HCAT, and each of their domains.

Variable	Previous knowledge				Internship duration				Marital status				Age		GPA	
		M ± SD	t	*p*		M ± SD	t	*p*		M ± SD	t	*p*	r	*p*	r	*p*
SEA	No	5.1 ± 1.6	0.58	0.563	8 m	4.7 ± 1.8	−1.32	0.194	M	4.7 ± 1.8	−0.89	0.383	0.07	0.361	**0.44**	< 0.001*
	Yes	4.9 ± 1.8			> 8 m	5.1 ± 1.6			S	5.1 ± 1.6						
OEA	No	5.1 ± 1.6	0.61	0.546	8 m	4.8 ± 1.7	−0.89	0.376	M	4.8 ± 1.7	−0.66	0.513	0.06	0.45	**0.51**	< 0.001*
	Yes	4.9 ± 1.8			> 8 m	5.1 ± 1.6			S	5.1 ± 1.6						
UOE	No	5 ± 1.7	0.3	0.768	8 m	4.8 ± 1.8	−0.86	0.396	M	4.7 ± 1.9	−0.93	0.359	0.07	0.327	**0.45**	< 0.001*
	Yes	4.9 ± 1.9			> 8 m	5.1 ± 1.7			S	5.1 ± 1.7						
ROE	No	4.8 ± 1.7	0.5	0.616	8 m	4.4 ± 1.7	−1.34	0.188	M	4.2 ± 1.6	−1.59	0.125	0.12	0.114	**0.4**	< 0.001*
	Yes	4.7 ± 1.8			> 8 m	4.9 ± 1.7			S	4.8 ± 1.7						
WLEIS	No	5 ± 1.5	0.53	0.601	8 m	4.7 ± 1.7	−1.15	0.256	M	4.6 ± 1.6	−1.09	0.286	0.09	0.252	**0.48**	< 0.001*
	Yes	4.9 ± 1.7			> 8 m	5 ± 1.5			S	5 ± 1.6						
PLENP	No	3.2 ± 0.9	1.5	0.137	**8 m**	**2.8 ± 1**	**−2.08**	0.043*	M	2.9 ± 1	−0.69	0.495	**0.25**	0.001*	**0.35**	< 0.001*
	Yes	2.9 ± 0.9			**> 8 m**	**3.2 ± 0.9**			S	3.1 ± 0.9						
MC	No	3.1 ± 0.9	1.3	0.196	8 m	2.8 ± 1	−1.75	0.087	M	3 ± 1	−0.54	0.595	**0.28**	< 0.001*	**0.28**	< 0.001*
	Yes	2.9 ± 0.9			> 8 m	3.1 ± 0.8			S	3.1 ± 0.9						
LNM	No	3.1 ± 0.9	1.48	0.143	**8 m**	**2.7 ± 1**	**−2.03**	0.048*	M	2.7 ± 1	−1.44	0.164	**0.25**	0.001*	**0.26**	< 0.001*
	Yes	2.8 ± 0.9			**> 8 m**	**3.1 ± 0.9**			S	3.1 ± 0.9						
PD	No	3.1 ± 0.9	1.76	0.082	8 m	2.8 ± 1	−1.68	0.1	M	2.9 ± 1.1	−0.7	0.488	**0.31**	< 0.001*	**0.28**	< 0.001*
	Yes	2.9 ± 1			> 8 m	3.1 ± 0.9			S	3.1 ± 0.9						
HCAT	No	3.1 ± 0.8	1.52	0.133	8 m	2.8 ± 1	−1.98	0.054	M	2.9 ± 1	−0.92	0.369	**0.27**	< 0.001*	**0.31**	< 0.001*
	Yes	2.9 ± 0.9			> 8 m	3.1 ± 0.8			S	3.1 ± 0.8						

Bold value indicate significant result. * indicates statistically significant at level *p* < 0.001.

### Structural equation model for the relationship between WLEIS and HCAT

The final model, which demonstrated improved fit indices [χ^2^/df = 2.23 (<5), CFI = 0.98 (>0.90), TLI = 0.97 (>0.90), RMSEA = 0.08 (<0.1), SRMR = 0.07 (<0.08)], indicating an overall acceptable model fit. In the measurement models, all WLEIS domains (SEA, OEA, UOE, and ROE) demonstrated significant loadings, with standardized coefficients ranging from 0.86 to 0.93, indicating strong contributions to the latent construct of EI. Similarly, the HCAT domains (PLENP, MC, LNM, and PD) showed significant loadings with standardized coefficients between 0.91 and 0.97, supporting their role as indicators of the clinical competence (Table 4).

**Table 4:** Structural equation model.

Models	Coefficient	SE	Standardized coefficient	*p*
Measurement models				
Emotional intelligence (WLEIS)				
SEA	1		0.90	< 0.001*
OEA	1.00	0.05	0.93	< 0.001*
UOE	1.08	0.05	0.93	< 0.001*
ROE	1.00	0.06	0.86	< 0.001*
Clinical competence (HCAT)				
PLENP	1		0.91	< 0.001*
MC	1.04	0.04	0.97	< 0.001*
LNM	1.07	0.04	0.95	< 0.001*
PD	1.05	0.05	0.94	< 0.001*
Structural model				
Clinical competence (HCAT)				
WLEIS	0.33	0.04	0.62	< 0.001*
Age	0.15	0.04	0.25	< 0.001*
Emotional intelligence (WLEIS)				
GPA	1.23	0.17	0.50	< 0.001*
Fit indices				
χ2/df	2.23			
CFI	0.98			
TLI	0.97			
RMSEA	0.08			
SRMR	0.07			

SE: standard error; χ2/df: Chi-square statistic per degree of freedom; CFI: Comparative Fit Index; TLI: Tucker Lewis Index; RMSEA: Root Mean Square Error of Approximation; SRMR: Standardized Root Mean Square Residual. * indicates statistically significant at level *p* < 0.001.

**Figure 1:**
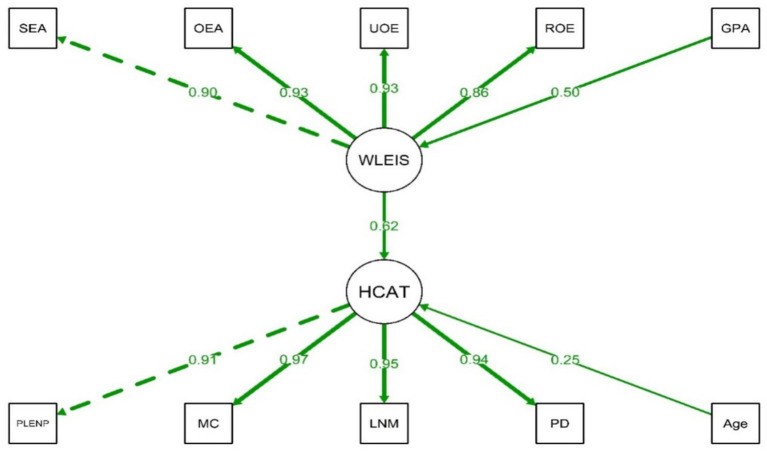
Structural equation model depicting the relationships between emotional intelligence (WLEIS) and the clinical competence (HCAT).

In the structural model, EI (WLEIS) had a significant positive effect on the clinical competence (HCAT) with a standardized coefficient of 0.62. Age was also positively associated with the clinical competence (HCAT) (standardized coefficient = 0.25), while GPA showed a significant association with EI (WLEIS) (standardized coefficient = 0.50). The structural model is illustrated in Figure 1. The model demonstrates both direct and indirect pathways through which EI influences nursing interns’ clinical competence, highlighting the mediating roles of age and GPA within the overall structural framework.

## Discussion

This study examined the relationship between EI and nurse interns’ clinical competence, as well as its association with selected socio-demographic characteristics. The findings indicated that EI was significantly associated with nursing interns’ clinical competence, supporting the growing body of evidence emphasizing the critical role of EI in nursing education and clinical practice. Despite several studies reporting an association between EI and the clinical competence, this relationship can vary depending on internship structure and clinical context (18, 23), highlighting the importance of context-specific considerations.

Sociodemographic factors, an internship duration of more than eight months was significantly associated with higher scores in the leadership and nursing management domain of clinical competence While this is consistent with previous research suggesting that clinical leadership skills among newly graduated nurses develop progressively during the first year of practice (24), other research has reported variability in intern’s readiness and competency development during clinical rotation, including leadership skills (25).

Leadership competence is a critical component of nursing practice, as effective leadership behaviors contribute to improved patient outcomes, including patient satisfaction, safety, and reduced mortality (26). Clinical exposure during internship may therefore provide nursing interns with opportunities to enhance leadership skills, develop decision-making skills, and application of different leadership styles (27).

The present study also revealed a significant positive association between GPA and all domains of EI. This finding aligns with previous studies indicating that stronger academic performance is associated with higher levels of self-awareness, self-regulation, and EI (20). A high GPA is linked to a higher perception of clinical preparation requirements (28). It is explained by the fact that nursing students perform better academically because of their positive personality traits, which are based on their levels of self-understanding and self- regulation (29). Furthermore, the current study found that GPA was associated with the emotion regulation domain, consistent with prior findings. The present findings extend this by suggesting that GPA may indirectly influence clinical competence through its relationship with all domine of the EI. Alshammari et al. (30) also highlighted that nurse interns with higher GPAs tend to demonstrate strong critical thinking when assessing and addressing patients’ health issues.

In the current study, age was associated with the clinical competence This finding is consistent with previous research by Alshammari et al. (30). Similarly, Ye et al. (31) reported that older and longer practicum students tend to have greater knowledge and experience in clinical nursing tasks and skills. These students are likely to adopt avoidance and more confident in seeking and utilizing the necessary support (31). In contrast, younger nursing students may demonstrate lower self-efficacy due to limited experience and reduced self-confidence (32), which might affect their clinical competence. However, Fauji and Ruspitasari (33) stated that demographic factors, such as age were not significantly associated with t the quality of the internship experience. Instead, factors like mentorship quality and the work environment were more important predictors (33). This difference may be recognized by variations in study settings, sample characteristics, or other contextual factors in the working environment.

In the current study, prior knowledge of EI was positively associated with the professional, legal and ethical nursing practice domain of clinical competence. This finding is consistent with previous evidence indicating that EI training enhances emotional response and behavioral response among students (34). Such training in EI components may improve the efficiency of nursing care services and professional competence due to reduced stress levels (34). Accordingly, integrating EI into the curriculum of universities (35). Nurses with higher levels of EI are generally better able to manage ethical dilemmas, regulate emotional responses, and make appropriate professional judgments in complex situations and under pressure.

Professional, legal and ethical nursing practice refers to the ability to understand right from wrong related to the nursing field, which deals with people and their lives (36). In the current study, previous knowledge of emotional intelligence was positively associated with the professional, legal and ethical nursing practice (PLENP) domain of clinical competence Supporting this, Nouri and Dehghani (37) found that nurses with higher emotional and moral intelligence tend to demonstrate can make more logical, and ethical decisions focused on their goals despite their emotions and feelings. Further, research indicated that emotional and moral intelligence in nurses are associated with increased clinical competence e (38).

Based on the final fitted model, EI was significantly associated with the clinical competence of nursing interns, which is consistent with Rankin (39). Previous studies have also shown that EI is a significant predictor of success in clinical practice and academic performance (40, 41). Tehrani et al. (35) highlighted that students should appropriately develop their emotional regulation and general cognitive abilities. Previous studies have been indicated that there is a positive significant relationship between EI and problem-solving skills (42), critical thinking (43), and empathy (44). Additionally, Parkero et al. (45) indicated a strong correlation between the dimensions of EI with academic success and professional competence. Other studies suggested that EI is a crucial component of nursing care and positively influance the standard of care, involving the importance of spiritual intelligence (46, 47). These findings suggest that higher EI may improve clinical competence among interns by enhancing their ability to understand their deep emotions and express them naturally, regulating their emotions, using their emotions to guide their performance, and perceiving and understanding the emotions of people around them.

Therefore, to strengthen nursing care competencies among nursing interns, educational content aimed at developing and improving spiritual intelligence and EI must be incorporated into the foundation modules with curricula. Educators should emphasize the development of nursing students’ spiritual intelligence and EI throughout their training process. In this study, mediation analysis revealed that an internship duration of more than 8 months was significantly associated with the professional, legal and ethical nursing practice (PLENP) and leadership and nursing management (LNM) domains of competence. The ability to recognize and make appropriate resolutions to ethical problems requires a high level of ethical sensitivity, defined as the ability to distinguish ethical problems (48, 49). Low levels of ethical sensitivity have been associated with reduced the quality of patient care, decreased trust in nursing services, and lower job satisfaction (50). Therefore, it is crucial for nurses to improve their ethical sensitivity skills through education and training. Pervious evidence suggests that ethical sensitivity can be developed through education and reinforced by the ethical codes of the profession (51). Another study found that intern nurses had a significantly higher level of ethical sensitivity (52). These findings indicate that incorporating ethical principles, professional codes, and values into nursing curricula may enhance students’ ethical sensitivity and improve students’ preparedness to address ethical dilemmas or problems in their professional lives (52).

Collectively, these findings support the importance of EI as a critical component of clinical competence during nursing internships. EI skills appear to improve nursing interns’ ability to navigate complex clinical situations, manage emotional demands, and perform effectively across multiple domains of practice. Integrating EI into nursing education and internship programs may therefore contribute to improved clinical readiness and professional performance.

This study has some limitations: first, the use of a cross-sectional design limits the ability to establish causal relationships between EI and clinical competence. Second, the study sample include Saudi nursing interns, which may restrict the generalizability of the results to non-Saudi populations or nursing interns in different cultural or healthcare contexts. Further, convenience sampling was employed in this study, which may introduce selection bias and further limit the representativeness of the sample. Therefore, future studies using more diverse samples and probability sampling methods are recommended to enhance generalizability.

A clear limitation of this study relates to the use of a single, self-report online survey to collect both emotional intelligence (WLEIS) and clinical competence (HCAT) data simultaneously. This design introduces the possibility of common method bias, as participants evaluated both their own emotional abilities and their clinical competence in the same sitting. Such an approach may inflate the observed association between variables, which is particularly relevant given the relatively large reported coefficient (*β* = 0.62). The absence of independent or supervisor-based assessments of clinical competence further limits the ability to validate these findings. Future research should address this issue by incorporating multi-source data, such as supervisor evaluations or objective performance measures.

### Implications for nursing education and practice

The findings of this study have crucial implication for nursing education and internship programs in Saudi Arabia. The significant association of EI and the clinical competence suggests that nursing curricula in Saudi schools of nursing should integrate structured EI training to enhance students’ clinical performance and patient care outcomes. Furthermore, internship programs in Saudi healthcare settings should integrate EI focused workshops and reflective practices to support interns in managing both their own emotions and those of their patients.

Although age was not significantly associated with clinical competence, the findings highlight the critical role of mentorship and the clinical learning environment, which is consistent with previous studies (31, 33). Therefore, nursing schools and healthcare organizations in Saudi Arabia should invest in structured training programs for clinical preceptors to enhance their teaching and supervisory skills to both newly joined and more experienced nurse interns. This particularly important given evidence that younger interns may be more vulnerable to burnout and secondary traumatic stress (31).

Furthermore, the positive association between internship duration and leadership and nursing management competencies indicates the need to strengthen leadership training, simulation exercises, and opportunities for role delegation within the curriculum. Similarly, internship programs should offer workshops and guided clinical experiences that allow intern to practice leadership, critical thinking, and resource management skills in real healthcare environments (53, 54).

## Conclusion

This study examined the relationship between EI and nurse interns’ clinical competence, and its association with socio-demographic factors. In this study, EI directly associated with nurse interns’ clinical competence. Additionally, this study demonstrated that prior knowledge and training in EI have a statistically significant positive association with enhanced EI and clinical competence. Hence, it is crucial to integrate EI into the nursing curriculum as well as to develop nursing interns’ leadership skills to prepare them well for nursing practice.

Although integrating EI as a core component in the nursing curriculum can offer long-term benefits, yet requires extensive planning and resources, which may yield challenging of immediate implementation. As a practical alternative, a short, evidence-based EI training program for nursing interns before the commencement of the internship program is recommended. Grounded in validated frameworks, using structured assessments and innovative teaching strategies can enhance readiness, support clinical performance, and provide further data to guide curricular integration.

## Data Availability

The raw data supporting the conclusions of this article will be made available by the authors, without undue reservation.
